# Emergent dynamics of cellular decision making in multi-node mutually repressive regulatory networks

**DOI:** 10.1098/rsif.2025.0190

**Published:** 2025-08-20

**Authors:** Harshavardhan BV, Hanuma Sai Billakurthi, Sarah Adigwe, Kishore Hari, Herbert Levine, Tomas Gedeon, Mohit Kumar Jolly

**Affiliations:** ^1^ IISc Mathematics Initiative, Indian Institute of Science, Bengaluru, Karnataka, India; ^2^ Department of Bioengineering, Indian Institute of Science, Bengaluru, Karnataka, India; ^3^ Department of Mathematical Sciences, Montana State University, Bozeman, MT, USA; ^4^ Center for Theoretical Biological Physics, Northeastern University, Boston, MA, USA; ^5^ Department of Physics, Northeastern University, Boston, MA, USA

**Keywords:** gene regulatory network, Boolean modelling, mutually repressive networks, multi-lineage differentiation

## Abstract

Stem cell differentiation during development is governed by the dynamics of the underlying gene regulatory networks (GRNs). Mutually inhibiting nodes/collection of nodes encompass the GRNs that govern differentiation to two distinct fates. However, the properties of GRNs that can allow differentiation into n-terminal phenotypes are poorly understood. In this study, we examine toggle-n networks, encompassing mutual inhibitions among multiple transcription factors (TFs), to derive generalized insights regarding the dynamics underlying differentiation into n-terminal phenotypes. We show through numerical and analytical methods that steady-state distributions of these networks involve co-expression of multiple cell state-specific TFs, indicating the presence of multi-potent hybrid phenotypes during multi-lineage differentiation. Furthermore, incorporating a case study of T-helper cell differentiation, we show that cytokine signalling and specific asymmetry of regulatory links can drive further directed differentiation of these hybrid phenotypes into particular cell states within our mathematical framework.

## 1. Introduction

Elucidating the design principles of cell fate decisions has profound implications for understanding developmental biology, regenerative medicine and synthetic biology [1]. The process of cell fate determination can be conceptualized through the metaphor of Waddington’s landscape, illustrating the myriad trajectories a cell can undertake during differentiation. Each valley in this landscape represents a terminally differentiated phenotype defined by specific gene expression patterns and underlying epigenetic structures. These trajectories could contain straight paths corresponding to uni-potent lineages or branches, which represent the event of differentiation of a precursor or progenitor cell to the sister lineages to which it could give rise (usually two or three) [2,3]. These trajectories are finely regulated by the dynamics of gene regulatory networks (GRNs), which orchestrate gene expression patterns and shape the landscape, thus guiding cells towards specific fates [4,5]. These GRNs often exhibit multistability, which allows them to adopt any of these distinct stable states, where each corresponds to different cellular phenotypes [6,7]. Central to this paradigm is the concept of master regulators, the cell state-specific transcription factors (TFs) that are highly expressed exclusively to a specific lineage [8]. This conceptualization highlights the precision in the commitment of a cell to a particular lineage, marked predominantly by the activity of the corresponding master regulator. It should be noted that (terminally) differentiated cells could show plasticity and switch to another cell state, which may not be apparent from the term ‘terminal’ [9]. Such phenomena would also warrant the Waddington landscape not to be static, as traditionally believed, but rather dynamically influenced and altered by signalling pathways, epigenetic modifications and environmental cues [10]. Cell state-specific TFs for more than one particular cell type tend to be co-expressed in certain scenarios; for instance, T-bet and GATA3 inhibit each other but can also be stably co-expressed [11], and the TFs specific to other cell states (such as other T-helper states) are excluded.

Cell-fate choices between two and three possible states are well studied using a toggle switch (GRN featuring mutual inhibition between two TFs) and a toggle triad (GRN featuring mutual inhibition between three TFs) through ordinary differential equation (ODE)-based formalism [12–16] and Boolean approaches [17–19]. Also, the role of extracellular signalling in directing differentiation trajectories has been elucidated using dynamical systems approaches, particularly for a toggle switch [20,21]. However, a stem cell can differentiate into more than three cell fates; for example, a hematopoietic stem cell can differentiate into various terminal cell types, including erythrocytes, basophils, eosinophils, neutrophils, megakaryocytes, monocytes and lymphocytes [2,22]. The dynamics of networks that may enable more than two or three possible cell states need to be analysed further.

**Figure 1.**
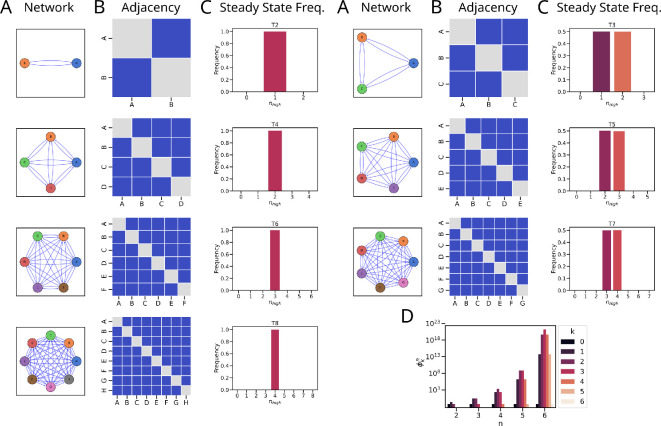
Schematic representation of the Tn networks and their steady states. (A) Graphical network representation illustrating the nodes and edges representing TFs and interactions, respectively. (B) Adjacency matrix representation with the rows/columns and elements representing the interaction, respectively. Red/+1, blue/−1 and grey/0 represent activatory, inhibitory and no interactions. (C) Steady-state frequency distribution of $n_{\text{high}}$ states. Left: even networks. Right: odd networks. (D) Number of monotone Boolean functions (see §4.5 for details) that support *k*-high states for networks with *n* nodes.

Recent studies have ventured into larger GRNs with mutual inhibition, the toggle tetrahedron (featuring mutual inhibitions between four TFs), where single positive states corresponding to the terminally differentiated phenotypes were not the predominant ones, necessitating additional mechanisms for complete differentiation [23,24]. Whether this pattern is an anomaly or a consistent trend with larger networks requires further investigation. In this study, we extend these findings by examining networks featuring mutual inhibitions, specifically toggle-n (Tn) networks, where *n* denotes the number of nodes, to derive generalized insights regarding the dynamics underlying differentiation into n terminal phenotypes. We analyse such networks with a varying number of nodes, ranging from 2 to 8 (figure 1A,B), using two complementary approaches. On the one hand, we simulate the Boolean dynamics of these networks until convergence using the Ising update formalism [25], and we enumerate the steady states that these iterations reach. On the other hand, we generate all possible Boolean update functions that are compatible with the network structure and directly (i.e. without simulation) enumerate how many of them support what type of steady state. The update functions that are compatible with the network structure are *monotone* with respect to the signs of the edges, i.e. for negative edges, they are decreasing (see §4.5 for precise definitions). The fact that results obtained by these complementary approaches agree gives us confidence that the structure of the network, rather than a particular type of regulatory logic, is responsible for them.

We find that in steady-state distributions of these models, TFs are co-expressed from state-specific TFs of multiple states, suggesting the presence of multi-potent hybrid phenotypes during multi-lineage differentiation. These traits hold true even under random variations in regulatory link strengths and the influence of other genes. Additionally, we demonstrate in our mathematical framework how factors such as cytokine signalling and specific asymmetry of regulatory links can drive further directed differentiation of these hybrid phenotypes into particular cell states. Overall, our results suggest that while Tn networks could play a pivotal role in differentiation, they necessitate additional mechanisms to effectively orchestrate the trajectories towards (terminal) cell fates.

## 2. Results

### 2.1. Mutually repressive regulatory networks allow the cell to bifurcate into precursor lineages

In our investigation, we analysed the steady-state distributions arising from Tn networks using asynchronous Boolean simulations with the Ising formalism; details are presented in the §4. We observed distinct frequency patterns of $n_{\text{high}}$, i.e. the number of nodes/TFs that are ON (high) in individual steady states for these different-sized networks. For the networks with two (T2), four (T4), six (T6) and eight (T8) nodes, we obtained 1 high, 2 high, 3 high and 4 high states, respectively. In other words, for a T6 network (i.e. six nodes mutually inhibiting one another), the steady state(s) show any three out of six nodes being ON (high) and the remaining three being OFF (low). The patterns for networks with three (T3), five (T5) and seven (T7) nodes have the frequencies shared between 1 high–2 high, 2 high–3 high and 3 high–4 high, respectively (figure 1C). In other words, for a T5 network (i.e. five nodes mutually inhibiting one another), the steady state(s) show any two or three out of five nodes being ON (high) and the remaining three or two being OFF (low). Since these networks are symmetric, the frequencies of the individual states with the same $n_{\text{high}}$ are equal. Furthermore, the frequency of states with $n_{\text{high}}$ equals the frequency of states with $n_{\text{high}}^{\prime }=n-n_{\text{high}}$.

We confirm these numerical results by analysing the convergence properties of Ising update function dynamics. Based on ideas from [25], we show that the states with configuration k≈n/2 (where k represents the number of high nodes) exhibit the lowest energy within the system. Furthermore, the Ising update ensures a strict decrease in the energy function for any state distinct from the states with the lowest energy. As a consequence, these states with k≈n/2 act as attractors in the Ising update dynamics. These findings are summarized in the following theorem, whose detailed proof can be found in electronic supplementary material, §S.1.

**Theorem 2.1*****.** Ising model update for network* Tn *for any* n≥2*, applied to any initial condition will converge to a steady state with* k *high states, where*


$$k=\left\{ \begin{array}{ll} \frac{n}{2} & n\text{ is even }\\ \frac{n-1}{2}\text{ or }\frac{n+1}{2} & n\text{ is odd }. \end{array}\right.$$

To investigate whether the choice of the Ising update function or the repressive structure of the networks is responsible for the distribution of the steady states, we explicitly constructed the set of all MBF that are compatible with each of the networks Tn for n=2,3,4,5,6. Any function $f:B^{k}\to B$ with B:={0,1} is a *Boolean function*. A Boolean function f is increasing (decreasing) with respect to ith input if the increase in ith value from 0 to 1 does not decrease the value of f (decrease in ith value from 1 to 0 does not increase the value of f). The function f is a *monotone Boolean function* if it is either increasing or decreasing with respect to all of its inputs. Finally, a collection of MBFs $f=\left(f_{1},f_{2},\ldots ,f_{n}\right)$ where each $f_{i}$ is the update function of the state $x_{i}$ associated with network node i of network N, is *a monotone Boolean function $f:B^{n}\to B^{n}$ for network* N . We construct the set of all these MBFs for the networks Tn for n=2,3,4,5,6 and examine the steady states they support. In particular, we computed the number of MBFs ($\phi_{k}^{n}$) that support steady states with k=0,1,…,n high states in network Tn (figure 1D). These numbers are listed in the ascending order below:


$$\begin{array}{rl} T2 & :(1,4,1)\\ T3 & :(1,45,45,1)\\ T4 & :(1,4104,38416,4104,1)\\ T5 & :(1,(20^{4})(167),(84^{3})(148^{2}),(84^{3})(148^{2}),(20^{4})(167),1)\\ T6 & :(1,(168^{5})(7580),(2008^{4})(7413^{2}),(5573^{6}),(2008^{4})(7413^{2}),(168^{5})(7580),1). \end{array}$$

The number of MBFs $f:B^{s}\to B$ of s variables is the Dedekind number D(s) that grows extremely quickly and is only known for s≤9 [26,27]. Since the number of MBF functions $f:B^{n}\to B^{n}$ for the network Tn is a product of Dedekind numbers over the number of nodes n, the number of MBFs grows even more quickly. Therefore, we were unable to compute the steady-state prevalence for T7 and T8.

Note that these numbers confirm the observations from the Ising update studies cited above and suggest that the network structure, rather than the choice of the Ising update, is responsible for these results. The methodology is described in detail in §4.5.

These systems are also multi-stable in nature; perturbing a steady state by flipping one node from ON to OFF or vice versa can lead to a switch to one of the other possible steady states. For even values of *n* in Tn networks, the majority of the perturbations go back to their original state; for odd value of *n*, however, the perturbation can lead them to adopt a state at a Hamming distance of 1 (electronic supplementary material, figure S1).

Introducing self-activation and self-inhibition mechanisms into the networks had notable effects on steady-state patterns. The pattern remains unchanged upon the addition of self-activation for 3- (T3SA), 5- (T5SA) and 7-node (T7SA) networks. However, a distinct pattern is seen for even values of *n*. For the 2-node network (T2SA—a toggle switch with self-activation), while 1 high states continue to be most dominant, 0 high (both nodes are OFF) and 2 high (both nodes are ON) also appear. Similarly, for 4- (T4SA), 6- (T6SA) and 8-node (T8SA) networks, the combinations of 1 high and 3 high, 2 high and 4 high and those of 3 high and 5 high appear too (electronic supplementary material, figure S2). Conversely, upon the addition of self-inhibition, the pattern remains the same for 2- (T2SI), 4- (T4SI), 6- (T6SI) and 8-node (T8SI) networks. However, for the 3- (T3SI), 5- (T5SI) and 7-node (T7SI) networks here, no steady states were observed (electronic supplementary material, figure S3). When examining the state transitions graphs in these networks, we observe that the states that were stable (i.e. there were no outgoing edges to other nodes) in the cases without self-regulation are able to transition to other previously observed stable states while maintaining their self-edges. We will call such states ‘meta-stable’ (electronic supplementary material, figure S4A,B). The new transitions occur because the self-inhibition on the nodes that are low/off facilitates them to transition to states where they are high. However, in the case of even networks with self-inhibition, the inhibition from the nodes that are high could not be overcome by this mechanism, rendering these states stable (electronic supplementary material, figure S4C).

**Figure 2.**
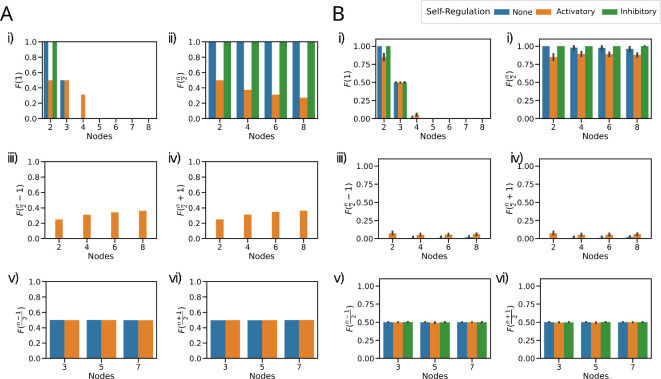
Steady-state distributions are dominated by states with *n*/2 nodes high. (A) Frequency of *k*-high state (F(k)) for (i) F(1) single node high; (ii) F(n/2) nodes high for even networks; (iii) F(n/2−1) nodes high for even networks; (iv) F(n/2+1) nodes high for even networks; (v) F((n−1)/2) nodes high for odd networks; and (vi) F((n+1)/2) for odd networks. (B) Frequency of *k*-high state (F(k)) for Tn networks with random edge weights ∈U(0,1). (i) F(1) single node high; (ii) F(n/2) nodes high for even networks; (iii) F(n/2−1) nodes high for even networks; (iv) F(n/2+1) nodes high for even networks; (v) F((n−1)/2) nodes high for odd networks; and (vi) F((n+1)/2) for odd networks. The error bars represent the 95% confidence interval.

Next, we have looked at how the frequency of states with k-high nodes, F(k), changes with the number of nodes in the network. Thus, F(1) would indicate the terminal phenotypes with one TF active. It follows from theorem 2.1 that F(1) drops sharply from 1 for 2 nodes to 0.5 for 3 nodes and 0 for 4 nodes and more, i.e. single-positive (1 high) states are not observed for networks beyond the toggle triad. The addition of self-activation was able to rescue F(1) only for the 4-node network (figure 2A). The direct relevance of theorem 2.1 is evident when we consider $F(\frac{n}{2})$ for networks with an even number of nodes; the value is exactly 1 for the networks with no self-regulation and for networks with self-inhibition. The networks with self-activation lose some $F(\frac{n}{2})$ to the states with Hamming distance 1, i.e. the frequency of ‘flanking states’ $F(\frac{n}{2}-1)=F(\frac{n}{2}+1)≠0$ . Interestingly, the frequency of these ‘flanking states’ seems to increase with increasing number of nodes (figure 2A). Similarly, it follows from theorem 2.1 that the networks with an odd number of nodes have equal values of 0.5 for $F(\frac{n-1}{2})$ and $F(\frac{n+1}{2})$.

Overall, the $\approx \frac{n}{2}$ high steady states are predominant for n node mutually repressive regulatory networks instead of single-positive ones that denote (terminally) differentiated states. This could indicate that such networks could govern the differentiation into precursor lineages or progenitor phenotypes with more than three terminal phenotypes. Other regulatory mechanisms would then, therefore, be required for the cell to fully differentiate into terminal phenotypes.

### 2.2. Steady-state patterns are robust to edge perturbations

Our analysis so far implicitly provides equal weighting to each edge in the network. To assess the resilience of observed steady-state patterns against extrinsic biological noise [28], we introduced some variations to the edge weights, reflecting heterogeneity in the strength of regulatory interactions (electronic supplementary material, figure S5A). Specifically, we sampled the magnitude of each regulatory link from a uniform distribution U(0,1)*,* deviating from the fixed value of 1. We generated 100 such unique sets of edge-weight values, each combination denoting a different set of values for varied strength of regulatory interactions among different nodes in that network.

Interestingly, despite these perturbations, the overall pattern of steady states remained largely unchanged, with only minor variations observed. For instance, while the frequency of F(1) showed only minor differences compared to the equal-weighted case, notable differences included T4 and T3SI. The pattern becomes more clear when looking at $F(\frac{n}{2})$, $F(\frac{n}{2}-1)$ and $F(\frac{n}{2}+1)$. The states with $\frac{n}{2}$ high are more frequently observed in random-weighted networks compared to equal-weighted networks of the same size with self-activation. In contrast, minor variations could occasionally push the $\frac{n}{2}$ states to the states with Hamming distance = 1 (‘flanking’ states) for the networks without self-regulation. Similarly, the patterns observed in $F(\frac{n-1}{2})$ and $F(\frac{n+1}{2})$ remained consistent, albeit with slight variations, in networks without self-regulation as well as in networks with self-activation (figure 2B). Interestingly, in circuits with self-inhibition, we observed that the system converges to specific steady states, unlike the case with equal-weighted edges. In certain scenarios, the random sampling chooses weights that do not allow for transition between some of the ‘metastable’ states and thus stabilizing them (electronic supplementary material, figure S4B,D,E).

**Figure 3.**
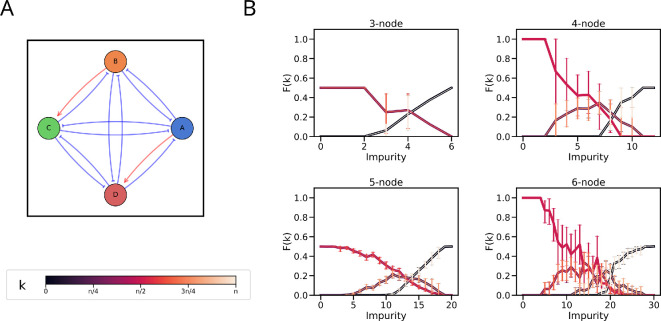
Edge-sign perturbations maintain the steady-state distributions for low levels of impurity. (A) Schematic of edge-sign perturbation for a 4-node network with two impurities. (B) Frequency of *k*-high state (F(k)) for (i) 3-, (ii) 4-, (iii) 5- and (iv) 6-node networks where each inhibition is replaced by activation (Impurity). The colour of each line corresponds to the *k* as given in the colour bar, with *n* denoting the number of nodes. The error bars represent the 95% confidence interval.

We then explored if perturbations of the sign of regulation would have any effect on the steady-state distributions (figure 3A). For a low extent of ‘impurity’, i.e. the replacement of an inhibition by an excitation, the F(2) and F(3) corresponding to $F(\frac{n}{2})$ for 4-node and 6-node networks maintain their frequency of 1. Likewise, the F(1)–F(2) and F(2)–F(3) corresponding to $F(\frac{n-1}{2})$–$F(\frac{n+1}{2})$ for 3-node and 5-node networks maintain their frequency of 0.5 each for low impurity (figure 3B), suggesting that the system dynamics exhibits functional resilience to minor changes in network topological space.

As the extent of impurity increased, the steady-state frequency patterns began to change. For even values of *n*, the frequency distribution shifts to $F(\frac{n}{2}-1)$–$F(\frac{n}{2}+1)$ and then broadens to $F(\frac{n}{2}-2)$–$F(\frac{n}{2}+2)$, while for odd values of *n*, it shifts to $F(\frac{n-2}{2})$–$F(\frac{n+2}{2})$. Finally, as expected, F(0) and F(n) become the most dominant states with a frequency of 0.5 each at the maximum level of impurity (i.e. where all inhibitions have been replaced with activation). However, F(1) does not exceed 0.5, irrespective of the extent of impurity.

In summary, despite perturbations in edge weights and signs of regulation, steady-state patterns of Tn networks exhibit robustness, with only minor variations observed, suggesting resilience to intrinsic biological noise.

### 2.3. Mutually repressive regulatory networks maintain their behaviour when embedded in random networks

In biological systems, genes seldom operate in isolation but rather form complex interaction networks. Although, for the sake of simplicity, we have assumed the genes (nodes) of the Tn network do not interact with any other gene except those in the network, it should be noted that they only form a small part of a larger network comprising interconnected signalling and regulatory elements. We wanted to understand how Tn networks behave within such intricate biological contexts and whether these networks would show resilience to represent differentiation [29]. Without going into the details of specific signalling pathways in the larger network, we embedded them (see §4.2.3) into 100 random networks of varying embedding size (i.e. number of nodes in the random network) and embedding density (i.e. average number of edges per node in the random network) (electronic supplementary material, figure S5B).

**Figure 4.**
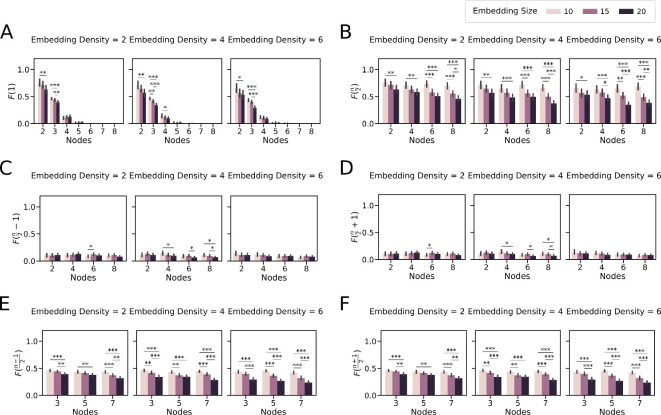
Steady-state distributions of the toggle networks are preserved when embedded in random networks: frequency of *k*-high state (*F*(*k*)) for networks embedded in random networks. The colours represent embedding size (number of nodes in the random network), and the columns represent embedding density (average number of edges in the random network). (A) *F*(1) single node high. (B) *F*(*n*/2) nodes high for even networks. (C) *F*(*n*/2−1) nodes high for even networks. (D) *F*(*n*/2+1) nodes high for even networks. (E) *F*((*n*−1)/2) nodes high for odd networks. (F) *F*((*n* + 1)/2). The error bars represent the 95% confidence interval. The asterisks denote the significance level of *t*‐test: ∗p<0.05;∗∗p<0.01;∗∗∗p<0.001.

We observed that the fundamental behaviours of Tn networks remain largely unchanged when integrated into these random networks. We observed a consistent pattern where the frequency of F(1) decreased as the number of nodes in the mutually repressive regulatory network increased (figure 4A), similar to the observations for these networks under isolated conditions. Notably, T4, T5 and T6 deviated from this pattern by displaying non-zero F(1) values, though relatively low. The frequency of $F(\frac{n}{2})$ remained the highest for even networks, followed by the states at a Hamming distance of 1 (figure 4B–D). Similarly, for odd networks, $F(\frac{n-1}{2})$ and $F(\frac{n+1}{2})$ maintained the highest frequency (figure 4E,F). Interestingly, we noted that the frequency of these dominant states $F(\frac{n}{2})$, $F(\frac{n-1}{2})$ and $F(\frac{n+1}{2})$ reduced with increasing embedding size and, to a lesser extent, with increasing embedding density.

Despite the random interaction among networks, the overall outcomes of Tn networks remain consistent with that observed in isolation. It is also worth noting that we did not control for any specific network architecture. For example, a network interacting in a hierarchical manner, i.e. different levels of organization corresponding to each differentiation event, with the Tn networks could potentially influence their behaviour in a directed manner [30,31].

### 2.4. Network of mutually inhibiting teams of transcription factors mirror the toggle networks

To check if these simplified Tn networks represent the behaviour of larger networks where a group of TFs regulates a particular phenotype, we considered ‘Team-*n*’ networks. These are fully connected networks with *n* teams such that the nodes within a team have activatory edges and nodes across teams have inhibitory edges (electronic supplementary material, figure S5C). A team $T_{i}$ *is* said to be high when the expression of all the members in a team is high.

**Figure 5.**
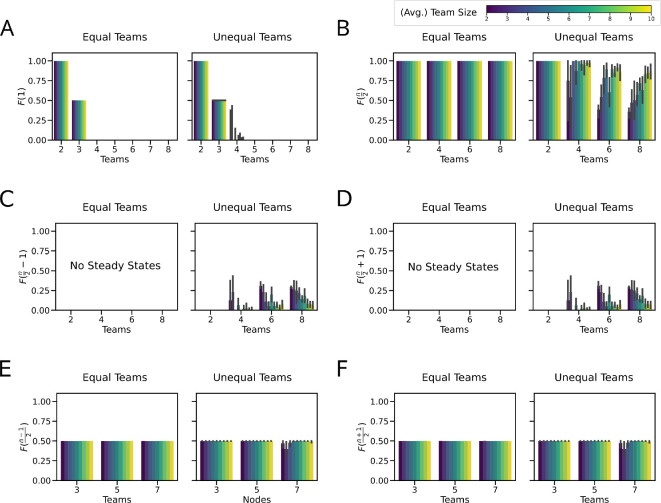
Steady-state distributions of mutually repressing team network mirror the toggle networks: frequency of *k*-high state (*F*(*k*)) for Team-*n* network of varying team sizes. Left column: team sizes are equal for all teams. Right column: team sizes are different across teams. The colours represent the average team size. (A) *F*(1) single node high. (B) *F*(*n*/2) nodes high for even networks. (C) *F*(*n*/2−1) nodes high for even networks. (D) *F*(*n*/2+1) nodes high for even networks. (E) *F*((*n*−1)/2) nodes high for odd networks. (F) *F*((*n* + 1)/2). The error bars represent the 95% confidence interval.

As a start, we assumed the number of members (m) to be equal between the teams. The F(1) matches the exact same frequency of the Tn networks regardless of the number of members (figure 5A). Comparing the $F(\frac{n}{2})$, $F(\frac{n}{2}-1)$ and $F(\frac{n}{2}+1)$ for the networks with even number of teams with those of Tn networks, we see that the pattern matches with the networks with either no regulation or self-inhibition (figure 5B–D). Similarly, comparing $F(\frac{n}{2}-1)$ and $F(\frac{n}{2}+1)$ for odd networks narrows it down to match the Tn case without self-regulation (figure 5E,F).

However, there can be cases where the number of members in a particular team can vary across teams [32]. So, we have also considered cases by randomly splitting the members between the teams. The F(1) in this case, while dropping with an increasing number of teams, also shows a non-zero frequency with the four-team network compared to the equal teams case (figure 5A). However, this frequency shows a high variance between the networks considered, and the frequency also drops with increasing average team size m. Similarly, while the behaviour for even networks remained largely unchanged, with the $\frac{n}{2}$ states still being the dominant states, the states with a Hamming distance of 1 from it ($\frac{n}{2}-1$ and $\frac{n}{2}+1$) seem to steal some of the frequency away from it for low average team sizes, again with a high variance (figure 5B–D). The $\frac{n-1}{2}$ and $\frac{n+1}{2}$ states also remain similar to the team-*n* case for networks with an odd number of teams (figure 5E,F). This is reminiscent of the perturbation of edge weights, and the fraction of members in a particular team could be considered equivalent to the relative strength of interactions between the teams. The high variance for networks with a low average team size could be attributed to the larger differences in team sizes, which create more discrete levels. As the average team size increases, we expect these F(k) values to converge to the levels seen with edge-weight perturbations, because the differences in team size become smaller, resulting in more continuous levels.

These results show that Tn networks can be used to represent larger networks of TFs interacting in a team-like manner.

### 2.5. Multi-level formalism reveals hybrid states characterized by intermediate expression levels

After thoroughly examining the phenotypic spaces of ‘Team-*n*’ networks, we asked if expanding the state space of the simulation formalism would unveil any additional characteristics of these networks. We simulated fully connected Team-n networks with equal number of members in each team, using a set of multi-level models defined by equation (4.4). Briefly, the multi-level formalism expands the state space by increasing the number of expression levels each node can have. For example, in the four-level model, node expressions take four values −{−1,−0.5,0.5,1} instead of two {-1,1} *in* the traditional Boolean formalism. To characterize the steady states of the multi-level model, we used team scores (average expression of all nodes in a given team). As the nodes in each team showed identical expression levels, the team score perfectly reflects the expression of every node in the team (electronic supplementary material, figure S6).

**Figure 6.**
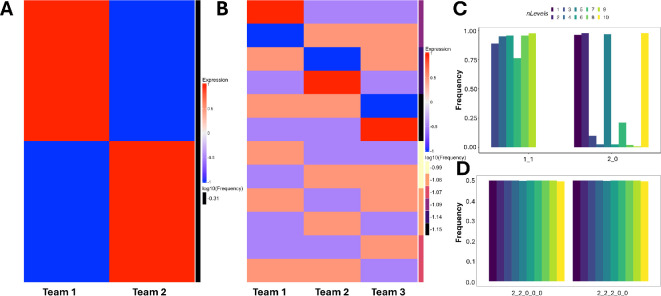
Steady-state resultant from multi-level formalism mirror the toggle networks but with partial team scores. (A) Team scores for the steady states of two-team network simulated using four-level model. Each column corresponds to a team and each row corresponds to a steady state. The red–blue colour scale represents the team score, i.e. the average expression of all nodes in a team. The yellow–black colour scale represents the ${log}_{10}(Frequency)$ of the corresponding steady state. (B) Same as (A), but for three-team network. (C) normalized and discretized steady-state configurations for two-team network for different levels of the multi-level formalism. Each point on the *x*-axis represents the normalized form of a group of steady states. For example, 1_1 represents the steady states where both teams show partial expression. The *y*-axis shows the total frequency of the group of steady states represented by the normalized form. (D) Same as (C) but for five-team network.

Recently, we found that simulating GRNs underlying epithelial–mesenchymal plasticity (EMP) using four levels uncovers new hybrid states (states where nodes from both E and M teams have non-zero expression) in the phenotypic landscape [33]. However, simulations of the two-team network using a four-level model revealed no new steady states (figure 6A). While EMP networks are also two-team networks, some nodes do not completely obey the team structure, causing ‘frustration’ in the network structure, a property absent in the two-team network simulated here. A network is structurally frustrated if there exists at least one pair of nodes in the network such that not all the direct and indirect interactions in the network are of the same sign. Note the in two-team network, every pair of nodes belonging to the same team is connected by only positive direct and indirect (mediated by other nodes in the network) interactions and vice versa. Therefore, the two-team network is not frustrated. Because such frustration can lead to an increase in the fraction of hybrid states [34,35], we attribute the lack of new hybrid states in the two team networks to the lack of frustration. While a two-team network is not frustrated, every Team-*n* network with n>2 is frustrated. Consider three nodes *A*, *B* and *C* in a three-team network, each belonging to a different team. Since the networks are fully connected, these nodes mutually inhibit each other. Considering the interactions between *A* and *B*, the direct interactions both have a negative sign, but the indirect interaction mediated by *C* consists of two connected inhibitions resulting in a net positive interaction. This reasoning holds true for any pair of nodes in Team-n networks (n>2). Thus, we expect to see new hybrid states when Team-n networks are simulated using the multi-level formalism.

In contrast to a two-team network, a three-team network results in an entirely new set of steady states when simulated with multi-level models. While the configuration of states remained the same (high-low-low and high-high-low; figure 6B), all steady states of the three-team network show partial expression in at least two out of three teams. As the number of levels increases, all teams show partial expression in all steady states. The maximum expression level of each node also decreases as the number of levels increases (electronic supplementary material, figure S7A). This decrease in expression level was observed across networks with 4–10 teams (electronic supplementary material, figure S7).

We then asked if the partial expression of teams is purely because of the decrease in the maximum possible node expression. We normalized the expression of each node by dividing the expression level by the maximum possible expression in the simulation and categorized the team scores into 0, 2 or 1, corresponding to a normalized expression of −1, 1 or between −1 and 1, respectively. For two- and three-team networks, we found that most steady states have partial team expression, even after the normalization, such that the steady-state configuration with the highest frequency was 11 for the two-team network and 111 for the three-team network (figure 6C and electronic supplementary material, figure S8A). We found similar dominance of partial expression in the four-team network as well (electronic supplementary material, figure S8B). However, from the five-team onwards, we find that even with more levels in the allowed state space, all team scores were either 2 or 0 (figure 6D and electronic supplementary material, figure S8C–F). More than 99% of the state space converged to the steady states expected from the team configurations (22000 and 22200 for five-team networks, 222000 for six-team networks, and so on).

### 2.6. Toggle-n networks require the synergy of epigenetic reprogramming and cytokine signalling to adopt terminal fate

To further investigate the mechanisms that cells can adopt for making robust cell fate choices, we introduced asymmetry in the strength of transcriptional links in the networks. This asymmetry could arise from variations in DNA methylation, histone acetylation or mRNA degradation [36]. We focus on one of the nodes in the network, the TF A, and explore if such mechanisms can push towards a state where *A* is expressed exclusively (single positive *A* state). Therefore, we examine the frequency of the single positive state where only TF A is highly expressed, $F_{A}(1)$. We hypothesized that stronger inhibition of TF A on the other cell state-specific TFs would direct cells towards a cell state characterized by high expression of TF A.

**Figure 7.**
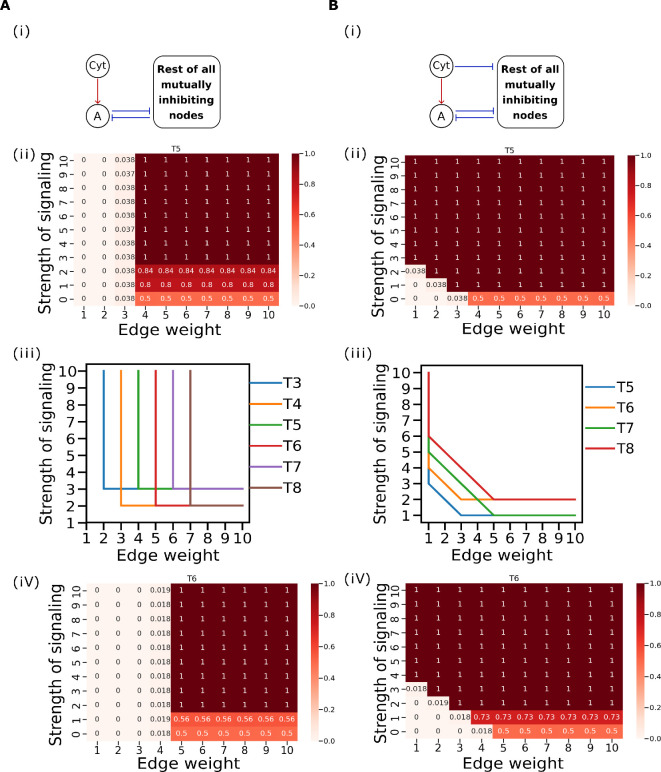
Relative frequency of single positive *A* high **$F_{A}(1)$** with increasing strength of signalling and edge weight. (A) (i) Network diagram representing cytokine (Cyt) activating the TF A. (ii) Heatmap representation of the relative frequency of the cell state $F_{A}(1)$ with increasing edge weight and strength of signalling for a representative case of toggle pentagon. (iii) Line plot showing the threshold values of the strengths of signalling for a range of values of edge weights and the threshold values of edge weights for a range of values of strengths of signalling for observing $F_{A}(1)$ with a relative frequency of 1. (iv) Heatmap representation of the relative frequency of the cell state $F_{A}(1)$ with increasing edge weight and strength of signalling for a representative case of toggle hexagon. (B) Same as (A) but for the scenario in which the cytokine inhibits other TFs in addition to activating TF A.

We increased the inhibitory link strengths from *A* to all remaining nodes (referred to edge weights hereafter) from 1 to 10, while keeping the rest at a default value of 1, and analysed the steady-state distributions of cell states. We expected that higher edge weights would be able to have $F_{A}(1)=1$. However, we observe that the steady-state distribution of $F_{A}(1)$ saturates at 0.5, with the remaining states exhibiting co-expression of other cell state-specific TFs along with *A* (figure 7A).

Next, we investigated the role of cytokine signalling in cellular differentiation. We hypothesized that a cytokine that transcriptionally activates *A* would push the cells towards the single positive *A* high state. We introduced a cytokine that constitutively activates *A* in our simulations and varied the strength of signalling (strength of activation of *A* by the cytokine) from 0 to 10 and measured $F_{A}(1)$ as before. Contrary to our expectations, we observed no significant changes in the distribution of the cell state with increasing signalling strength (figure 7A).

We then combined increased edge weights (corresponding to epigenetic reprogramming) with varying strengths of cytokine signalling and ran the simulations again. The results suggest that a threshold level of both signalling strength and edge weight is required for complete differentiation towards a single positive *A* high state. This means that both cytokine signalling and epigenetic reprogramming are necessary simultaneously, and one cannot compensate for the absence or deficiency of the other. The threshold for edge weight increases linearly with the size of the network. For a network with three nodes (T3), the edge weight threshold is 2; for T4, it is 3; for T5, it is 4; for T6, it is 5; for T7, it is 6; and finally T8, where the threshold is 7. In contrast, the threshold for signalling strength only depends on whether the network has an odd or even number of nodes. For networks with an odd number of nodes (T3, T5, T7), the signalling strength threshold is 3, whereas for the networks with an even number of nodes (T4, T6, T8), the threshold is 4 (figure 7A).

Finally, we have included inhibitory edges from the cytokine to other nodes, keeping the existing activatory link on *A* to see if this scenario would be able to give only single positive *A* high state. Consistent with our hypothesis, we observed only the *A* high state ($F_{A}(1)=1$) in the steady-state distribution with a sufficient increase solely in the strength of signalling (both strength of activation on *A* by the cytokine and strength of inhibition on the other nodes by the cytokine). The signalling strength required for $F_{A}(1)=1$ in this case increases linearly with network size. For networks with five nodes (T5), the signalling strength threshold is 3; for T6, it is 4; for T7, it is 5; and for T8, the threshold is 6 (figure 7B). Similarly, in this scenario, increased edge weight can compensate for the deficiency of signalling strength and the signalling strength threshold for $F_{A}(1)=1$ decreases with increasing edge weight (figure 7B).

### 2.7. Case study: T-helper cell differentiation ($T_{h}1/T_{h}2/T_{h}17/T_{reg}/T_{FH}$ decision making)

Our results so far consider simplified toy models (Tn networks). We next investigated where Tn structures could be present in real biological networks identified so far. Here, we present a case study of T-helper cell or CD4${,}^{+}$ T-cell differentiation and examine the evidence for the existence of Tn networks.

There are several types of CD4${,}^{+}$ T cells, T-helper 1 ($T_{h}1$), T-helper 2 ($T_{h}2$), T-helper 17 ($T_{h}17$), regulatory T cells ($T_{reg}$) and T follicular helper ($T_{FH}$) to name a few. All of them are derived from the same lineage of naive CD4${,}^{+}$ T cell ($T_{h}0$), and each has a distinct role in both innate and adaptive immunity and secrete specific cytokines. They are also characterized by the master regulators that are exclusively expressed in each of the cell types, $T_{h}1$: T-bet (TBX21); $T_{h}2$: GATA3; $T_{h}17$: RORγt (RORC); $T_{reg}$: FOXP3; and $T_{FH}$: BCL6 [37].

**Figure 8.**
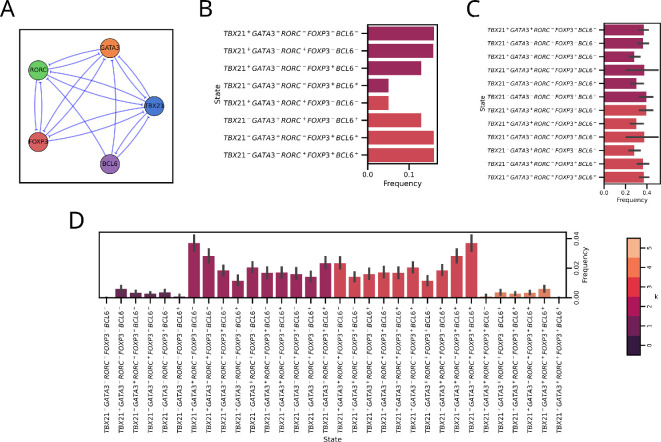
T-helper cell differentiation network is an incomplete mutually inhibitory network which shares properties of T5 network. (A) Graphical network representation illustrating the nodes and edges representing master regulators and regulatory interactions, respectively. (B) Steady-state distribution of the 5-node T-helper cell network. (C) Steady-state distribution of the 5-node network with random edge weights ∈U(0,1). (D) Steady-state distribution of the 5-node network embedded in random networks. The error bars represent the 95% confidence interval. The colour of each bar corresponds to the number of high nodes (k) as given in the colour bar.

T-bet, GATA3, RORγt and FOXP3 have been reported to mutually inhibit one another [23]. Similarly, BCL6 and T-bet can inhibit each other [38,39]. BCL6 is also reported to inhibit GATA3 and RORγt, thus preventing differentiation of $T_{h}1$, $T_{h}2$ and $T_{h}17$ phenotypes [40–43]. However, no direct evidence has yet been reported for BCL6 being inhibited by GATA3, RORγt or FOXP3. Therefore, while this network is not exactly a T5 network, but given that 16 out of 20 possible interactions ($2\times {,}^{5}C_{2}$) are mutually inhibitory in nature, we proceeded to analyse this incomplete T5 network as a case study (figure 8A and electronic supplementary material, table S1).

The differentiation between $T_{\text{h}}1–T_{\text{h}}2$ and $T_{\text{h}}17–T_{\text{reg}}$ are classic examples modelled using the Toggle Switch, featuring mutual inhibition between T-bet–GATA3 and RORγt–FOXP3 [44–46]. Similarly, previous studies have also modelled the differentiation event between $T_{\text{h}}1$, $T_{\text{h}}2$ and $T_{\text{h}}17$ using a 3-node mutually inhibitory network of T-bet, GATA3 and RORγt and between $T_{\text{h}}1$, $T_{\text{h}}2$, $T_{\text{h}}17$ and $T_{\text{reg}}$ using a 4-node mutually inhibitory network of T-Bet, GATA3, RORγt and FOXP3 [23,24,36]. These would correspond to T2, T3 and T4 networks, respectively (figure 1).

We simulated the 5-node incomplete network and observed a similar pattern to T5 with the 2 high and 3 high steady states, although, due to asymmetry, the frequencies between all the individual states are not equal, as in the case of Tn networks. Among the states with two nodes high, we observe the $T_{\text{h}}1/T_{\text{h}}2$ hybrid (TBX21${,}^{+}$ GATA3${,}^{+}$ RORC${,}^{-}$ FOXP3${,}^{-}$ BCL6${,}^{-}$) and $T_{\text{h}}1/T_{\text{h}}17$ hybrid (TBX21${,}^{+}$ GATA3${,}^{-}$ RORC${,}^{+}$ FOXP3${,}^{-}$ BCL6${,}^{-}$) to be the most dominant, followed by $T_{\text{h}}1/T_{\text{reg}}$ (TBX21${,}^{+}$ GATA3${,}^{-}$ RORC${,}^{-}$ FOXP3${,}^{+}$ BCL6${,}^{-}$) hybrid and $T_{\text{reg}}/T_{\text{FH}}$ hybrid (TBX21${,}^{-}$ GATA3${,}^{-}$ RORC${,}^{-}$ FOXP3${\;}^{+}$ BCL6${,}^{+}$). Among the states with three nodes high, we observe the $T_{\text{h}}17/T_{\text{reg}}/T_{\text{FH}}$ (TBX21${,}^{-}$ GATA3${,}^{-}$ RORC${,}^{+}$ FOXP3${,}^{+}$ BCL6${,}^{+}$) and $T_{\text{h}}2/T_{\text{reg}}/T_{\text{FH}}$ (TBX21${,}^{-}$ GATA3${,}^{+}$ RORC${,}^{-}$ FOXP3${,}^{+}$ BCL6${,}^{+}$) tri-hybrids to be the most dominant, followed by $T_{\text{h}}2/T_{\text{h}}17/T_{\text{FH}}$ (TBX21${,}^{-}$ GATA3${,}^{+}$ RORC${,}^{+}$ FOXP3${,}^{-}$ BCL6${,}^{+}$) and $T_{\text{h}}1/T_{\text{h}}2/T_{\text{h}}17$ (TBX21${,}^{+}$ GATA3${,}^{+}$ RORC${,}^{+}$ FOXP3${,}^{-}$ BCL6${,}^{-}$) (figure 8B).

Many master regulators are known to self-activate, reinforcing their activity in order to maintain their robustness in gene expression programs [7]. T-bet, GATA3, RORCγt and FOXP3 were reported to self-activate themselves [47–50]. By incorporating these known self-activatory edges in our network model (electronic supplementary material, figure S9A), we observed the emergence of $T_{\text{h}}2/T_{\text{FH}}$ (TBX21${,}^{-}$ GATA3${,}^{+}$ RORC${,}^{-}$ FOXP3${,}^{-}$ BCL6${,}^{+}$), $T_{\text{h}}17/T_{\text{FH}}$ (TBX21${,}^{-}$ GATA3${,}^{-}$ RORC${,}^{+}$ FOXP3${,}^{-}$ BCL6${,}^{+}$), $T_{\text{h}}1/T_{\text{h}}2/T_{\text{reg}}17$ (TBX21${,}^{+}$ GATA3${,}^{+}$ RORC${\;}^{-}$ FOXP3${\;}^{+}$ BCL6${,}^{-}$) and $T_{\text{h}}1/T_{\text{h}}17/T_{\text{reg}}$ (TBX21${,}^{+}$ GATA3${\;}^{-}$ RORC${,}^{+}$ FOXP3${\;}^{+}$ BCL6${,}^{-}$) states (electronic supplementary material, figure S9B). Similarly, simulating with random edge weights also sees the emergence of these states (figure 8C). Embedding the network shows all the 2 high and 3 high states (figure 8D). The states with 1 high and 4 high are also present, but their frequencies are negligible. In both the random edge weight and embedding cases, the variance observed in frequency is large, indicative of a different set of states appearing each time.

These results are in line with the current paradigm of CD4${,}^{+}$ T-cell plasticity where increasing emphasis is being given to the hybrid states [51,52]. Experimental evidence for hybrids states has been reported, such as $T_{\text{h}}1/T_{\text{h}}2$ hybrids [53], $T_{\text{h}}1/T_{\text{h}}17$ hybrid [54], $T_{\text{h}}1/T_{\text{reg}}$ [55], $T_{\text{h}}2/T_{\text{reg}}$, $T_{\text{h}}17/T_{\text{reg}}$ [56], $T_{\text{reg}}/T_{\text{FH}}$ [57,58], $T_{\text{h}}2/T_{\text{FH}}$ [59] and $T_{\text{h}}1/T_{\text{FH}}$, $T_{\text{h}}17/T_{\text{FH}}$ [60]. Although not all the tri-hybrid states are well characterized, states such as $T_{\text{h}}1/T_{\text{h}}2/T_{\text{reg}}$ [61] and $T_{\text{h}}1/T_{\text{h}}17/T_{\text{reg}}$ [62] have been found. Similarly, studies have also found T-helper cells expressing the cytokines corresponding to multiple cell types [63,64]. These hybrid states provide functionality for the differentiation to be gradual as well as for faster response to different stimuli [64,65].

The various cytokines produced by antigen-presenting cells drive differentiation of $T_{\text{h}}0$ into specific cell types. The cytokines that stimulate the differentiation towards the CD4${,}^{+}$ T cell mentioned above are as follows: $T_{\text{h}}1$ - IFNγ (interferon-gamma) and IL-12 (interleukin-12); $T_{\text{h}}2$ - IL-4 and IL-2; $T_{\text{h}}17$ - TGF-β (transforming growth factor-beta), IL-6 and IL-21; $T_{\text{reg}}$ - TGF-β and IL-2 [37].

**Figure 9.**
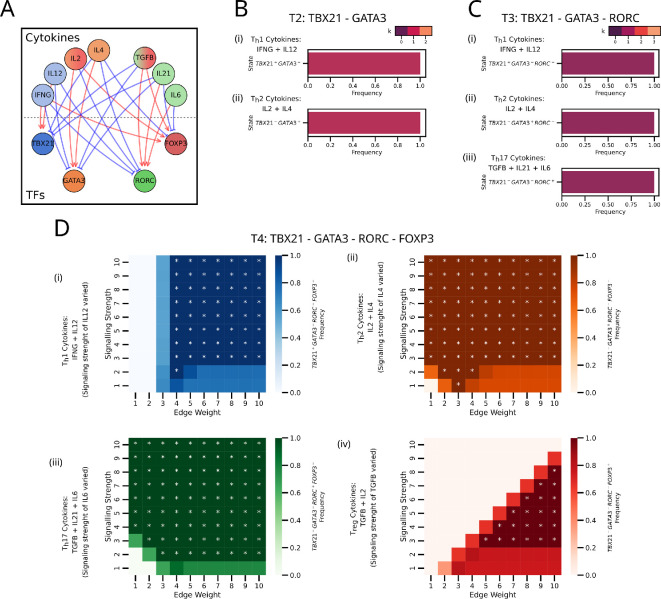
Cytokine signalling combinations can stimulate specific T-helper cell states. (A) Graphical network representation illustrating the nodes and edges representing cytokines and regulatory interactions, respectively. (B) Steady states of T2 network subject to (i) $T_{\text{h}}1$, (ii) $T_{\text{h}}2$ cytokines. (C) Steady states of T3 network subject to (i) $T_{\text{h}}1$,(ii) $T_{\text{h}}2$, (iii) $T_{\text{h}}17$ cytokines. (D) Steady-state frequencies of single-positive states, $F_{T_{h}i}(1)$ of (i) $T_{\text{h}}1$, (ii) $T_{\text{h}}2$, (iii) $T_{\text{h}}17$, (iv) $T_{\text{reg}}$ on changing edge weights and signalling strength for the respective cytokine combinations. The stars (*) denote when $F_{T_{h}i}(1)=1$.

We surveyed the literature for evidence of interaction of these cytokines with the master regulators (figure 9A and electronic supplementary material, table S2). We see a qualitative resemblance to the toy model where a cytokine activates their corresponding master regulators, while inhibiting others. This aligns with our earlier modelling (figure 7B), except for $T_{\text{reg}}$, which shares cytokines with $T_{\text{h}}2$ and $T_{\text{h}}17$, requiring these edges to be activatory.

We simulated the T2 (T-bet-GATA3), T3 (T-bet-GATA3-RORγt) and T4 (T-bet-GATA3-RORγt-FOXP3) networks with the exogenous cytokine combinations known to stimulate differentiation into a particular cell type. We have assumed that the cytokines are externally supplied and considered the autocrine and paracrine signalling to be negligible. For the T2 and T3 network, the presence of cytokines that activate one and inhibit the rest was sufficient; we did not need to invoke higher edge weights or signalling strength to undergo differentiation (figure 9B,C). However, for T4, higher edge weights or signalling strength were needed in addition to the cytokine effects to achieve differentiation, consistent with our results from the toy model (figure 9D). Specifically, $T_{h}1$ stimulation and $T_{\text{reg}}$ stimulation vary from the toy model. $T_{h}1$ stimulation requires a higher edge weight to overcome the activation from IFNγ to FOXP3. $T_{\text{reg}}$, on the other hand, has the side-effect of increased signalling activating RORγt, requiring a compensatory increase in edge weights from FOXP3 as well.

Together, these results indicate that synergy between signalling strength and regulatory strength is needed to achieve terminal T-helper cells with diverse phenotypes.

## 3. Discussion

Through our Boolean modelling approach, we aimed to unravel the dynamics of Tn networks and their ability to replicate the intricate process of cellular differentiation observed in biological systems. However, our findings suggest that these networks may not efficiently generate terminally differentiated states, through the frequency of single positive states F(1) or lack thereof. The predominance of ‘central states’, indicated by ≈n/2 for even networks with n *nodes* and (n−1)/2 - (n+1)/2 for odd networks with n *nodes,* suggests a bifurcating process rather than a direct path to terminal differentiation.

This bifurcating process may be more reflective of initial differentiation to precursor lineages or progenitor cells, which further differentiate to the terminal phenotypes [66]. For instance, in the case of haematopoietic stem cells, these can differentiate into progenitor cells committed to specific lineages, such as myeloid precursor and lymphoid precursor lineages. These lineages can further differentiate into erythrocytes, macrophages, neutrophils, basophils, eosinophils and platelets, and into B cells, T cells and natural killer cells, respectively [2,22].

Next, we went on to check on what sort of conditions these networks would be resilient to, and the conditions that can aid towards the terminal differentiation. We observe that when subjected to random noise through perturbation of the edges both in terms of their magnitude as well as sign, the networks maintained their traits. Similarly, the traits are also maintained when such networks interact with external signals from random networks. However, this behaviour changes when these mechanisms act in an asymmetric or biased manner. The presence of selective activatory external signal to one particular node (in this case *A*) or increased strength of inhibition from that node to other nodes are able to shift the stable states towards a single positive state involving that particular node $F_{A}(1)$. Additionally, these mechanisms act in a synergistic manner and contribute towards a higher $F_{A}(1)$ when acting in unison. These simple Tn GRNs also remain scalable when the TFs interact in a team-like manner as observed in various networks of cellular differentiation.

It is plausible that the actual GRN governing differentiation may exhibit a structure vastly different from what we have explored. Such GRNs can be inferred and constructed from experimental developmental data [67–69]. These GRNs could provide a more nuanced and biologically relevant understanding of the developmental process. However, our study has focused on a simplified scenario, where each cell state-specific TF inhibits every other, thereby scaling up the well-established motif of a toggle switch. However, the major assumptions and pitfalls of this modelling approach should also be acknowledged. We have assumed that all the TFs only have inhibitory interactions among themselves, as most of the interactions between these master regulators involved in binary cell-fate decisions tend to be inhibitory [2]. However, experimental evidence for Tn networks for *n* > 3 is not as strong as the toggle switch or triad case. Also, as the number of possible interactions (edges) increases as $O(n^{2})$ with *n*-phenotypes, comprehensive experimental testing may become infeasible. Instead, the indirect interactions between the TFs could be reduced to a single edge, albeit at the cost of missing out on secondary dynamics (e.g. delayed response) and intermediate regulations. This model also lacks detailed mechanisms of how the repression happens, whether it is direct transcriptional regulations, epigenetic modifications or by sequestering other genes [70,71], and considers only the presence or absence. Similarly, the edge weights in this model are not direct biological parameters but rather qualitative or relative measures. These weights represent an abstraction of the interaction strength of TFs, which can be challenging to translate back into precise biological terms.

Moreover, there is a possibility that the topology and strength of regulation within the GRN could be dynamic traits themselves. The regulations may not always be present and could be acting in a context-specific manner. While we have investigated discrete cases of remodelling regulatory strengths, it is conceivable that these regulations are subject to dynamic alterations and reinforcement through feedback mechanisms from specific nodes, including epigenetic feedback, for instance [36]. Nevertheless, such a comprehensive modelling effort falls beyond the scope of our current study.

Furthermore, the choice of a Boolean framework over a more continuous representation of states introduces its own set of implications. This choice was, however, justified due to the problems of defining a high state and discretizing the steady-state distributions for the context of our simulations. Even after relaxing the Boolean framework to allow more granular expression levels, we observe that the trends remain the same. However, it is worth noting that the undifferentiated stem-like state is not well defined. Even if we consider an all-high or all-low state to be representative of a stem cell, it is not a stable steady state. Moreover, the differentiation process is also a continuous process involving gradual transitions influenced by varying levels of TFs. Continuous frameworks such as ODEs are more suited to capture such dynamics [72].

The emergence of *n*/2 high stable states is a consequence of both the structure of the repressive networks we study and the Ising update function. We prove that the update dynamics of such a function always converge to one of the *n*/2 high stable states. It is plausible that alternative formalisms, such as those employing logical AND/OR gates, could yield vastly different results [73,74]. The selection of an appropriate Boolean function to represent regulations within mutually repressive networks remains a subject of debate. To address a concern about the generalizability of the reported results, we have also considered all possible MBFs compatible with signs of network edges and calculated the number of those functions that support a steady state with *k*-high states ($\phi_{k}^{n}$). Our calculations show that among all MBFs, the majority support the *n*/2 high steady state. However, this analysis cannot be done for larger networks as the number of MBFs (Dedekind number) grows extremely quickly. However, for large networks we can always analyse samples from the collection of all MBFs. On the other hand, we can replace Ising update model by selecting other MBFs, perhaps those which have high likelihood of occurrence in biological systems [75,76].

Our work provides valuable theoretical insight into the mechanistic aspects of directed differentiation of stem cells. Although we do not expect to see an exact match with experimental data given the oversimplifications made, the general phenomena should hold. While the presence of hybrid states is gaining acknowledgement from the experimental community [11,77], evidence for hybrid states involving the expression three or more state-specific TFs is minimal. Furthermore, the results from this study open up the debate about the concept of master regulators and whether a single TF is sufficient to determine the cell fate [78]. On that note, TFs that serve as master regulators for one set of lineages may also be expressed in different cell types of a different lineage under a different context. Although we have looked at only one specific case of T-helper cell differentiation, whether such structures exist within other complex differentiation events remains to be seen.

Similarly, this study can have profound implications in fields like synthetic biology and regenerative medicine. Synthetic biology can provide a controlled platform where the theoretical predictions of such systems can be tested under biological conditions. Recent advances in synthetic biology have allowed for the construction of multi-node gene circuits that exhibit multi-stable dynamics, which can be precisely manipulated and analysed [79,80]. The implications of Tn networks could be extended to the engineering of cells with desired functionalities for therapeutic purposes, such as engineered T cells for use in cancer immunotherapy.

## 4. Methods

### 4.1. Boolean formalism

We simulated the dynamics of the networks using asynchronous Boolean simulations. We used the Ising formalism, a well-established framework in physics [81] and which has been recently applied to the study of regulatory networks [17,25].

For a network with *n*-nodes, the state $x\in \{-1,+1{\}}^{n}$ is an *n*-dimensional vector. Here, $x_{i}=+1$ indicates the gene i to be in an ON/high state and $x_{i}=-1$ indicates the gene i to be in an OFF/low state. The choice of −1 over 0 for the low state is to allow a particular gene to contribute towards the regulation of the target nodes even in the low state.

The topology files which are the edges listed in tab-separated format were used as input for the simulation. During simulation, these were converted and represented as adjacency matrices Adj. The elements of Adj are such that:


(4.1) $$\begin{array}{r} {\text{Adj}}_{ij}=\left\{ \begin{array}{ll} +1 & \text{if }i\text{ activates }j\\ -1 & \text{if }i\text{ inhibits }j\\ 0 & \text{if no interaction from }i\text{ to }j. \end{array}\right. \end{array}$$

These simulations were performed by generating 100 000 initial conditions for each network topology. The updating was done using asynchronous update where k is chosen randomly in {1,2,⋯n} and the kth node is updated. This update was done for a maximum of 1000 time steps or until convergence to steady state. The update rules are as follows:


(4.2) $$\begin{array}{r} x_{j}(t+1)=\left\{ \begin{array}{ll} +1 & \text{if }\sum_{i}x_{i}(t)\cdot {\text{Adj}}_{ij}>0\;\&\;j=k\\ -1 & \text{if }\sum_{i}x_{i}(t)\cdot {\text{Adj}}_{ij}<0\;\&\;j=k\\ x_{j}(t) & \text{otherwise}. \end{array}\right. \end{array}$$

The simulation outputs the steady states and their average relative frequency f(i) across three replicates. The steady states that do not converge at the end of maximum time steps are not considered further. The frequency of k-high states F(k) is given by


(4.3) $$F(k)=\sum_{i\in K}f(i),\;\text{where}\;\;K=\{x|\sum_{i=1}^{n}(x_{i}=+1)=k\}.$$

We also simulated the Team-n networks with multi-level extension of the Ising formalism, governed by the following set of update rules:


(4.4) $$\begin{array}{r} x_{j}(t+1)=\left\{ \begin{array}{ll} -1 & \text{if }\frac{\sum_{i}{\text{Adj}}_{ij}x_{i}(t)}{d_{j}}<-\frac{l-1}{l}\\ -\frac{k}{l} & \text{if }-\frac{k}{l}\leq \frac{\sum_{i}{\text{Adj}}_{ij}x_{i}(t)}{d_{j}}<-\frac{k-1}{l},\;\forall k\in \{l-1,l-2,\ldots ,0\}\\ +\frac{k}{l} & \text{if }\frac{k-1}{l}<\frac{\sum_{i}{\text{Adj}}_{ij}x_{i}(t)}{d_{j}}\leq \frac{k}{l},\;\forall k\in \{1,2,\ldots ,l-1\}\\ +1 & \text{if }\frac{l-1}{l}<\frac{\sum_{i}{\text{Adj}}_{ij}x_{i}(t)}{d_{j}}\\ x_{j}(t) & \text{if }\frac{\sum_{i}{\text{Adj}}_{ij}x_{i}(t)}{d_{j}}=0, \end{array}\right. \end{array}$$

where l is the number of levels defining the state space’s granularity and $d_{j}$ is the in-degree of jth node.

### 4.2. Network generation

#### 4.2.1. Toggle-n networks

We generated networks using the NetworkX library [82] to create a series of Tn networks with varying number of nodes. Tn networks are mutually repressive regulatory networks, where each node inhibits the other nodes. To generate these networks, we created complete directed graphs for nodes ranging from 2 to 8 giving T2 to T8. The edge-weight parameter for all edges was set to 2, representing inhibition. For networks with self-regulations, self-edges are added to the graph with edge-weight parameter = 1 or edge weight = 2 for self-activation or self-inhibition cases, respectively. These were converted to edge-list format and saved as a tab-separated topology file (.topo file).

The adjacency matrix Adj(Toggle) in this case would be


(4.5) $$\begin{array}{r} {\text{ Adj }}_{ij}(\text{ Toggle })=\left\{ \begin{array}{ll} -1 & \text{ if }i\neq j\\ 0 & \text{ if }i=j\;\&\text{ Self-regulation }=\text{ None }\\ +1 & \text{ if }i=j\;\&\text{ Self-regulation }=\text{ Activatory }\\ -1 & \text{ if }i=j\;\&\text{ Self-regulation }=\text{ Inhibitory }. \end{array}\right. \end{array}$$

#### 4.2.2. Addition of impurities

We started with a particular Tn network and replaced $n_{\text{imp}}$ possible combination of inhibitory edges with activations for $n_{\text{imp}}\in 0,1,\cdots n(n-1)$. Note that many of the networks will be isomorphic due to the high symmetry of the Tn network. Recall that directed signed graphs G=(V,E) and $H(V^{\prime },E^{\prime })$ are isomorphic if there is a bijection $\varphi :V\to V^{\prime }$ such that if (v,w)∈E is a directed edge from v to w in E then $(\varphi (v),\varphi (w)\in E^{\prime }$ is a directed edge in $E^{\prime }$ with the same orientation and the same sign. Since many of the perturbed networks are isomorphic due to symmetry, we selected a maximum of 100 non-isomorphic graphs for n≤5 and 50 for n=6. For n>6, the number of combinations become too high and this analysis was not performed. The isomorphism testing was performed in a manner similar to that in the previous work [23]. For this, the complete directed-weighted graph was reduced into a directed-unweighted graph with the presence of an edge representing an activation and the lack representing an inhibition. The isomorphism was checked using nx.algorithms.is_isomorphic function which utilizes an implementation of the VF2++algorithm [83]. These were then saved as topology files.

#### 4.2.3. Embedding toggle networks in random networks

We embedded Tn networks in random directed graph networks. For each combination of embedding sizes (the number of nodes in the random network) of 10, 15 and 20, and embedding density (the average number of edges to each node) of 2, 4 and 6, 100 random directed graphs were generated using nx.gnm_random_graph. The edge weights for edges present in this network were randomly sampled from {−1,1}. This network was merged with each of the toggle networks considered. To connect the two networks together, all the edges between nodes of the random network and toggle network are randomly sampled from {−1,0,1}. These edges correspond to the interactions as defined in equation (4.1).

#### 4.2.4. Team-*n* networks

A Teams-n network consists of a directed graph with n-teams with nodes in each team (T) having intra-team activations and inter-team inhibitions. This is done by having an adjacency matrix Adj(Team), such that


(4.6) $$\begin{array}{r} {\text{Adj}}_{ij}\text{(Team)}=\left\{ \begin{array}{ll} +1 & \text{if }i\in T_{i},j\in T_{j},\&\;T_{i}=T_{j}\\ -1 & \text{if }i\in T_{i},j\in T_{j},\&\;T_{i}\cap T_{j}=\emptyset \;\\ 0 & \text{if }i=j. \end{array}\right. \end{array}$$

We have considered the cases of both equal members in each team and random unequal split of members between the teams. For the equal case, the number of members in a team, $n_{{Team}_{i}}=m$, where m∈{2,3,⋯10}. For the unequal case, 100 networks were generated where the $m_{i}$ *is* sampled from multinomial distribution with $\hat{m}\cdot n$ number of experiments and $p_{i}=1/n$, while ensuring that $m_{i}\neq 0$ for any i and $m_{i}\neq \hat{m}$ for all i.

Since, the steady states only allow all members of a team to be either all high or all low, the results would be consistent even with a weaker condition, such as average expression being greater than 0.5:


$$x_{T_{i}}=+1\;⟺\;x_{j}=+1\;\forall j\in T_{i}.$$

### 4.3. Edge-weight perturbation

To simulate the effect of biological noise acting through the strength of regulatory interactions, we considered each edge to have random weights. For each topology, we performed 100 sets of edge-weight sampling, where each element of the adjacency matrix was scaled by a randomly sampled variable from a uniform distribution between 0 and 1, U(0,1). This can be represented by the adjacency matrix Adj(E.W.):


(4.7) $${\text{Adj}}_{ij}(E.W.)=U(0,1)\cdot {\text{Adj}}_{ij}\text{(Toggle)}.$$

Further simulations were then performed as mentioned in §4.1.

Similarly, to assess the role of asymmetry of regulation strength in directed differentiation towards terminal fates, we altered the strengths W of inhibitory edges (referred to as edge weight hereafter) from node *A* to all remaining nodes when that node is high. In other words, if *A* is inhibiting *B* and has a edge weight of W>1, then node *A* would contribute −W, if *A* is high or +1, if *A* is expressed low to the net effect of transcriptional regulation of *B*. This weight W was varied from {1,2,⋯,10}. The adjacency matrix for such a case where *A* is the kth node is


(4.8) $$\begin{array}{r} {\text{Adj}}_{ij}\text{(Asymm)}=\left\{ \begin{array}{ll} {\text{Adj}}_{ij}\text{(Toggle)} & \text{if }i\neq k\\ {\text{Adj}}_{ij}\text{(Toggle)} & \text{if }i=k\;\&\;i=j\\ {\text{Adj}}_{ij}\text{(Toggle)} & \text{if }i=k\;\&\;i\neq j\;\&\;x_{i}=-1\\ W\cdot {\text{Adj}}_{ij}\text{(Toggle)} & \text{if }i=k\;\&\;i\neq j\;\&\;x_{i}=+1. \end{array}\right. \end{array}$$

The number of states with only *A* high, $F_{A}(1)$, was then quantified to assess the impact of this asymmetry in directed differentiation.

### 4.4. Signalling

Next, we wanted to decipher the role of synergy between cytokine signalling and asymmetry in the networks for directed differentiation towards $F_{A}(1)$. We have included a cytokine that activates cell state-specific TF A in the networks by making necessary changes in the topology file (§4.1) and performed simulations with increasing strength of signalling (similar to increasing W in equation 4.8) and edge weights. Suppose if the TF A is the kth node and the cytokine is in the lth node, then the adjacency matrix with an edge weight of W and a strength of signalling $W_{C}$ is given by


(4.9) $$\begin{array}{r} {\text{Adj}}_{ij}\text{(Asymm, Cytk)}=\left\{ \begin{array}{ll} W_{C} & \text{if }i=l\;\&\;j=k\\ 0 & \text{if }i=l\;\&\;j\neq k\\ 0 & \text{if }j=l\\ {\text{Adj}}_{ij}\text{(Asymm)} & \text{otherwise}. \end{array}\right. \end{array}$$

In addition to these, we included inhibitory edges on the rest of the nodes from the cytokine by keeping the existing activation on *A* intact. Now the adjacency matrix is given by


(4.10) $$\begin{array}{r} A_{ij}\text{(Asymm, ACytk)}=\left\{ \begin{array}{ll} W_{C} & \text{if }i=l\;\&\;j=k\\ -W_{C} & \text{if }i=l\;\&\;j\neq k\\ 0 & \text{if }j=l\\ {\text{Adj}}_{ij}\text{(Asymm)} & \text{otherwise}. \end{array}\right. \end{array}$$

### 4.5. Monotone Boolean functions

**Definition 4.1.** A function $f_{i}:B^{k}\to B$ is an *increasing (decreasing) monotone Boolean function* with respect to input $x_{j}$ if


$$b_{1}≺b_{2}\;\text{implies}\;f\left(b_{1}\right)\leq f\left(b_{2}\right)\;(f\left(b_{1}\right)\geq f\left(b_{2}\right)),$$

for all pairs $b_{1}≺b_{2}\in B^{k}$ which only differ in jth component and $b_{1}^{j}<b_{2}^{j}\in B$.

**Definition 4.2.** Consider a signed graph (network) of interactions N=(V,E) with a set of n vertices V and a set of edges E, where each edge e=e(i→j) has a sign $\delta_{i}^{j}=1\;(\delta_{i}^{j}=-1)$ for a *activating (repressing) edge*. A Boolean function $f=\left(f_{1},\ldots ,f_{n}\right)$ is a *monotone Boolean function* (*MBF) with respect to network N* [73,84] if each $f_{i}$ is monotone with respect to all its arguments $x_{j}$ that respect the sign $\delta_{i}^{j}$, i.e. $f_{i}$ is increasing Boolean function with respect to each $x_{j}$ where $\delta_{i}^{j}=1$ and decreasing Boolean function with respect to each $x_{j}$ where $\delta_{i}^{j}=-1$.

These definitions highlight that MBFs preserve the order of inputs, ensuring that an increase in input values does not result in a decrease in the function’s output.

#### 4.5.1. Formulation of the problem

Consider a series of networks Tn with n=2,3,4,5,6 with n nodes and repressive edges connecting all ordered pairs of nodes (i,j)∈V×V,i≠j. Let


$$E_{0}=(0,\ldots ,0),\;E_{1}=(1,0,\ldots ,0),\;E_{2}=(1,1,\ldots ,0),\;\ldots \;E_{n}=(1,\ldots ,1)$$

be states in $B^{n}$ with 0,1,…,n ones in the Boolean vector.

Consider a collection $F^{n}$ of MBFs monotone with respect to network Tn. An element of $F^{n}$ is a n-tuple $f=\left(f_{1},f_{2},\ldots f_{n}\right)$, where each $f_{j}:B^{n-1}\to B$ is a MBF that respects the signs of the network edges. Let $\phi_{k}^{n}$ be the number of MBFs $f\in F^{n}$ for which the state $E_{k}\in B^{n}$ is an equilibrium, i.e it satisfies


$$f_{j}(E_{k})=E_{k}^{j}\;\forall j,k.$$

Here $E_{k}^{j}$ is jth component of vector $E_{k}$.

Observe that since network Tn is invariant under the group of permutations $S_{n}$, the number of functions supporting equilibrum $E_{j}$ and an equilibrium obtained by any permutation of $E_{j}$, will be the same.

#### 4.5.2. Approach

**Example.** Before we describe how we evaluate numbers $\phi_{k}^{n}$, for n=3,4,5,6 and k=0,…,n, we illustrate our approach on a simple example of toggle switch [13] T2 with nodes 1,2 and corresponding Boolean variables $x_{1},x_{2}$. The MBFs with respect to T2 are pairs $f=\left(f_{1},f_{2}\right)$ where both $f_{1},f_{2}:B\to B$. There are three decreasing MBFs B→B and thus respect the signs of T2: the zero 0 (one 1) function, assigning to both inputs the output 0(1) and the −I function that assigns 0 to input 1 and 1 to input 0. Therefore $F^{n}$ has 3×3=9 functions $f=\left(f_{1},f_{2}\right)$. To compute $\phi_{0}^{2}$, the function $f=\left(f_{1},f_{2}\right)$ has to satisfy


$$f_{1}(0)=0\;\text{ and }\;f_{2}(0)=0.$$

There is exactly one such function f=(0,0). Similarly, to compute $\phi_{1}^{2}$ the function $f=\left(f_{1},f_{2}\right)$ has to satisfy


$$f_{1}(0)=1\;\text{ and }\;f_{2}(1)=0.$$

There are two functions {1,−I} that satisfy the first condition, and two functions {0,−I} that satisfy the second condition. This gives $\phi_{1}^{2}=2\times 2=4$. Finally, f=(1,1) is the unique function that supports $E_{2}$. Therefore,


(4.11) $$\begin{array}{lcr} & \phi^{2}:=(\phi_{0}^{2},\phi_{1}^{2},\phi_{2}^{2})=(1,4,1). & \end{array}$$

Even in this simple example, we observe a ‘hat’-like pattern where the equilibria with equal number of zeros and ones are supported by more MBFs than equilibria that are more uniform.

We compute $\phi_{k}^{n}$, for n=3,4,5,6 and k=0,…,n we proceed in three steps, which are implemented in Python:

(1) We explicitly construct the collection $F^{n},n=3,4,5,6$ by first constructing all Boolean functions $f_{i}:B^{n-1}\to B$, discarding those that are not decreasing and forming n-tuples of such functions $f=(f_{1},\ldots ,f_{n})\in F^{n}$.
(2) For any input $b\in B^{n-1}$, we calculate $u_{b}^{n}$ the number of Boolean functions $f_{i}:B^{n-1}\to B$ such that
$$f_{i}(b)=0,$$
  and its complement $v_{b}^{n}$, the number of functions $f_{i}:B^{n-1}\to B$, such that
$$f_{i}(b)=1.$$
  Clearly, $u_{b}^{n}+v_{b}^{n}=D(n-1)$ the Dedekind number [26,27] which is the number of all MBFs with n−1 inputs.
(3) The previous calculation is simplified by the observation that the MBF $f_{i}:B^{n-1}\to B$ is invariant under any permutation of the inputs. Therefore
f(b)=f(σ(b)) for any permutation of inputs σ.
  Therefore, the values $u_{b}^{n},v_{b}^{n}$ only depend on the number of 1s and 0s in the input b. We denote these values $u^{n}(j),v^{n}(j)$, where j=0,…,n−1 is the number of 1s in the input $b\in B^{n-1}$. These numbers are calculated by the Python code and are listed in table 1.

**Table 1.** Values of $u^{n}(j)$ and $v^{n}(j)$ for n=2,3,4,5,6.

Input in $B^{n-1}$	$u^{n}$	$v^{n}$
** n=2 **		
(0)	1	2
(1)	2	1
** n=3 **		
(0,0)	1	5
(1,0)	3	3
(1,1)	5	1
** n=4 **		
(0,0,0)	1	19
(1,0,0)	6	14
(1,1,0)	14	6
(1,1,1)	19	1
** n=5 **		
(0,0,0,0)	1	167
(1,0,0,0)	20	148
(1,1,0,0)	84	84
(1,1,1,0)	148	20
(1,1,1,1)	167	1
** n=6 **		
(0,0,0,0,0)	1	7580
(1,0,0,0,0)	168	7413
(1,1,0,0,0)	2008	5573
(1,1,1,0,0)	5573	2008
(1,1,1,1,0)	7413	168
(1,1,1,1,1)	7580	1

In the final step, we directly enumerate $\phi_{k}^{n}$ by using numbers $u^{n}(j)$ and $v^{n}(j)$. We illustrate this for the case n=4. We first compute $\phi_{0}^{4}$. The function $f=\left(f_{1},f_{2},f_{3},f_{4}\right)$ supports an equilibrium $E_{0}=\left(0,0,0,0\right)$ if


$$f_{1}(0,0,0)=0,\;f_{2}(0,0,0)=0,\;f_{3}(0,0,0)=0,\;f_{4}(0,0,0)=0.$$

Using the n=4 sub-table in table 1, we compute the number of functions that satisfy each of these conditions. Since any combination of such functions gives an MBF that supports $E_{0}$, we get


$$\phi_{0}^{4}=u^{4}(0)*u^{4}(0)*u^{4}(0)*u^{4}(0)=1*1*1*1=1.$$

A more interesting aspect is the computation of $\phi_{1}^{4}$. The function $f=\left(f_{1},f_{2},f_{3},f_{4}\right)$ supports an equilibrium $E_{1}=\left(1,0,0,0\right)$ if


$$f_{1}(0,0,0)=1,\;f_{2}(1,0,0)=0,\;f_{3}(1,0,0)=0,\;f_{4}(1,0,0)=0.$$

Using the n=4 sub-table in table 1, we compute


$$\phi_{1}^{4}=v^{4}(0)*u^{4}(1)*u^{4}(1)*u^{4}(1)=19*6*6*6=4104.$$

#### 4.5.3. Number of monotone Boolean functions supporting steady states in T3, T4, T5 and T6

##### 4.5.3.1. Network T3

We consider states $E_{0}=(0,0,0),E_{1}=(1,0,0),E_{2}=(1,1,0),E_{3}=\left(1,1,1\right)$. Then the numbers are


$$\begin{array}{rl} \phi_{0}^{3} & =u^{3}(0)u^{3}(0)u^{3}(0)=1*1*1=1,\\ \phi_{1}^{3} & =v^{3}(1)u^{3}(1)u^{3}(1)=5*3*3=45,\\ \phi_{2}^{3} & =u^{3}(1)u^{3}(1)u^{3}(2)=3*3*5=45,\\ \phi_{3}^{3} & =v^{3}(1)v^{3}(1)v^{3}(1)=1*1*1=1. \end{array}$$

In the vector form:


$$\phi^{3}=(\phi_{0}^{3},\phi_{1}^{3},\phi_{2}^{3},\phi_{3}^{3})=(1,45,45,1).$$

##### 4.5.3.2. Network T4

We consider states $E_{0}=(0,0,0,0),\;E_{1}=(1,0,0,0),\;E_{2}=(1,1,0,0),\;E_{3}=(1,1,1,0),\;E_{4}=(1,1,1,1).$ The numbers $\phi_{i}^{4}$ can be computed as above from $u^{4}(j),v^{4}(j)$ and give


$$\phi_{0}^{4}=\phi_{4}^{4}=1;\;\phi_{1}^{4}=\phi_{3}^{4}=19*6*6*6=4104;\;\phi_{2}^{4}=14^{4}=38\;416,$$

which in the vector form is


$$\phi^{4}=(1,4104,38\;416,4104,1).$$

##### 4.5.3.3. Network T5

We consider states $E_{0},E_{1},E_{2},E_{3},E_{4},E_{5}$. The numbers $\phi_{k}^{5}$ are


$$\phi_{0}^{5}=\phi_{5}^{5}=1,\;\phi_{1}^{5}=\phi_{4}^{5}=(20^{4})(167),\;\phi_{2}^{5}=\phi_{3}^{5}=(84^{3})(148^{2});$$

and in the vector form:


$$(1,(20^{4})(167),(84^{3})(148^{2}),(84^{3})(148^{2}),(20^{4})(167),1).$$

##### 4.5.3.4. Network T6

We consider states $E_{0},E_{1},E_{2},E_{3},E_{4},E_{5},E_{6}$. The numbers $\phi_{k}^{6}$ are


$$\phi_{0}^{6}=\phi_{6}^{6}=1,\;\phi_{1}^{6}=\phi_{5}^{6}=(168^{5})(7580),\;\phi_{2}^{6}=\phi_{4}^{6}=(2008^{4})(7413^{2}),\;\phi_{3}^{6}=(5573^{6});$$

and in the vector form:


$$(1,(168^{5})(7580),(2008^{4})(7413^{2}),(5573^{6}),(2008^{4})(7413^{2}),(168^{5})(7580),1).$$

## Data Availability

The codes used for simulations, scripts for analysis and the simulated data are available at Zenodo [85]. Electronic supplementary material is available online [86].
